# Assessment of psychosocial variables by parents of youth with type 1 diabetes mellitus

**DOI:** 10.1186/1758-5996-4-48

**Published:** 2012-11-22

**Authors:** Fani Eta Korn Malerbi, Carlos Antonio Negrato, Marilia B Gomes

**Affiliations:** 1 School of Psychology, Pontifícia Universidade Católica de São Paulo, Rua Monte Alegre 984, 05014 001, São Paulo, SP, Brazil; 2 Department of Internal Medicine, Bauru’s Diabetics Association, Bauru, SP, Brazil; 3 Department of Internal Medicine, Diabetes Unit, State University Hospital of Rio de Janeiro, Rio de Janeiro, RJ, Brazil

**Keywords:** Type 1 diabetes, Family functioning, Psychosocial variables, Diabetes in youth, Glycemic control

## Abstract

**Purpose:**

To evaluate the impact of type 1 diabetes (T1D) on family functioning and child-rearing practices from parents’ point of view, to assess parents’ health-related quality of life and to explore the relations between psychosocial variables and diabetes care outcomes in youth with diabetes.

**Methods:**

This research was part of the cross-sectional multicenter Brazilian Type 1 Diabetes Study, conducted between December 2008 and December 2010 in 28 public clinics of 20 cities across four Brazilian geographical regions. Psychosocial questions were addressed to 1,079 parents of patients with T1D through an interview (89.3% mothers, 52.5% Caucasians, 38.6 ± 7.6 years old). Overall, 72.5% of the families were from low or very low socioeconomic levels. Parents were also submitted to health-related quality of life instruments (EQ-5D+EQ-VAS). Clinical data from the last medical appointment were collected by a physician using standardized chart review forms. The demographic, educational and socioeconomic profiles were also obtained and HbA1c levels registered.

**Results:**

Discomfort and anxiety/depression were the main complaints in EQ-5D, and were significantly more frequent in mothers (37.3% and 53.4%, respectively) than in fathers (25.7% and 32.7%, respectively). The mother was the only parent involved in diabetes care in 50.5% of the cases. The majority of parents (78.5%) mentioned changes in family functioning after the diagnosis, although they neither treated their diabetic children differently from the others (76.3%), nor set prohibitions (69.1%) due to diabetes. The majority was worried about diabetes complications (96.4%) and felt overwhelmed by diabetes care (62.8%). Parents report of overwhelming was significantly associated with anxiety/depression, as measured by the EQ-5D questionnaire. Less than half of the patients had already slept over, and the permission to do it increased as a function of children’s age. Nearly half of the parents (52%) admitted to experiencing difficulties in setting limits for their children/adolescents. HbA1c levels in patients from this group (9.7 ± 2.5%) were significantly higher than those of children/adolescents whose parents reported no difficulties towards limit-setting (8.8 ± 2.1%). Parents whose children/adolescents reported the occurrence of hypoglycemic episodes in the last month complained significantly more about anxiety/depression (55.1%) than parents from patients who did not report it (45.7%). Also a significantly greater proportion of parents whose children/adolescents had been hospitalized due to hyperglycemia reported anxiety /depression (58.7%) than those whose children/adolescents had not been hospitalized (49.8%).

**Conclusions:**

After the diagnosis of T1D, the lifestyle of all family members changes, what interferes with their quality of life. Mothers are still the primary caregivers for children/adolescents with diabetes. Difficulty to set limits for children/adolescents may be a risk for poor metabolic control. The study demonstrates the importance of family context in the adjustment of young patients to T1D. The specific needs of T1D patients and their impact on a family routine must be considered for future improvement on therapy elements and strategies.

## Introduction

Type 1 diabetes mellitus (T1D) is a chronic condition with a rising incidence worldwide in developed as well as in developing countries including Brazil [1-3].

Being both a chronic and a progressive disease, diabetes is a challenge for children, adolescents and their parents as they need special support to keep it under control. The long-term benefits of early intensive glycemic control on the rate of chronic complications has been demonstrated by the Diabetes Control and Complications Trial (DCCT) and its long-term follow-up study, the Epidemiology of Diabetes Interventions and Complications (EDIC) Trial [4,5].

Diagnosis of diabetes, particularly T1D, represents a sudden and necessary requirement for the development of skills, in order to obtain a good glycemic control, because many responsibilities for the daily management of T1D are immediately assigned to the young patients and their families [6].

The treatment of T1D must be handled carefully to avoid or minimize the risk of serious short and long-term complications [4,7].

Although recent improvements in diabetes care offer more flexible treatment options, high rates of nonadherence to treatment regimens and less than optimal glycemic control have been consistently reported [8-10].

Several studies could not detect a clear association between more intensive regimens and improved glycemic control [11,12]. Data suggest that the ability and motivation of the patient and his/her family to manage diabetes must be guaranteed to achieve successful results [11].

The studies undertaken by the Hvidoere Study Group have examined in detail the psychosocial factors associated with metabolic control. Barriers to achieve good metabolic control were identified and quality of life (QoL) issues in youth with diabetes and their families were assessed [13]. Data suggest that diabetes care outcomes (as assessed by HbA1c) are improved in patients with a good QoL and in those who are supported by a stable, confident family unit with a clear agreement on sharing responsibilities towards diabetes management and control [14].

When diabetes is diagnosed in childhood, parents are those who must initially adapt themselves to the new condition and learn the daily management skills, in order to take most of the treatment responsibilities [15]. Indeed, after the diagnosis of diabetes the whole family must rethink its lifestyle, as the patient needs regular mealtimes and a balanced food intake according to the blood glucose levels and physical activity performed [16].

As children become older, generally the parental role decreases. During adolescence, many of the diabetes-related tasks can interfere with patients’ needs for independence and peer acceptance [16].

The transition from adolescence to adulthood is also a critical time when family support is necessary, as chronic diabetes complications may already develop and need regular screening [17].

Recently, the DAWN Youth Web Talk Study [18] investigated the attitudes, wishes and needs of young patients with T1D and of parents/carers of children and adolescents with T1D from eight countries. Major results showed that almost half of parents/caregivers reported poor well-being, the majority of them feeling overwhelmed by their child’s diabetes at least sometimes. They frequently worried about their child’s long-term health problems and hypoglycemic episodes. Most parents also reported having some disruption of their own work and other activities because of their child’s condition. Because that study did not collect clinical data it was not possible to establish the relationship between psychosocial data and youth’s metabolic control. Brazil was one of the participating countries of the DAWN Youth Web Talk Study, but the Brazilian samples were predominantly from the Southeast region - 75.5% out of 278 patients aged 18–25 years and 74.1% out of 421 parents of children/adolescents aged less than 18 years [19] - where the socioeconomic conditions are among the best of the country [20].

The present research was planned as part of the Brazilian Type 1 Diabetes Study in order to evaluate the impact of T1D on family functioning and child-rearing practices from parents’ point of view, to assess parents’ health-related quality of life and to explore the relations between psychosocial variables and diabetes care outcomes.

To our knowledge, this research constitutes the first national report in Brazil with such a large sample of T1D patients and their parents addressing demographic, clinical and psychosocial variables.

### Research design and methods

This research was part of an observational, cross-sectional, nationwide multicenter study conducted between December 2008 and December 2010 in 28 public secondary and tertiary care clinics^a^ of 20 cities across four Brazilian geographic regions (north/northeast, mid-west, southeast and south). All patients received health care from the National Brazilian Health Care System (NBHCS). To be eligible, the participant centers were required to have a diabetes clinic with at least one endocrinologist. Overall, 31 public clinics were identified and invited to participate. A short questionnaire was sent to the clinics to acquire information on the characteristics of the patient population, especially the number of T1D patients under routine care; three clinics did not send back the answered questionnaires until the implementation of the study and were not included. The inclusion criteria, which were determined from a standardized medical chart review form data, included the diagnosis of T1D by a physician based on typical clinical presentation, and the need to use insulin continuously since the diagnosis without interruption and medical follow-up for at least six months at the respective center.

The Brazilian Diabetes Society (BDS) coordinated the study by monitoring and reviewing all study-related documents and approving all amendments, questionnaires and publications. The questionnaires were constructed and reviewed by leading diabetologists and psychologists in Brazil before the final approval. Each center had a coordinator who was trained on how to perform the interview and analyze data obtained from the medical charts.

At each participating center, a physician collected data from the T1D patients' last clinical visit using standardized chart review forms and invited the accompanying parents to participate in the study. Eighty-five parents refused to participate (7.3%). There were no center differences in the refusal rate to participate in the study.

The demographic, educational and socioeconomic profiles were obtained and HbA1c levels were registered. HbA1c goals were defined as <8.5% for patients aged 0–6 years, <8% for patients aged 7–12 years, <7.5% for individuals 13–19 years old and <7% for patients aged at least 20 years [21]. The patients who had diabetes for more than five years were evaluated for the presence of chronic complications through review of medical chart forms within one year of the assessment, based on the performance of fundoscopy, microalbuminuria and a foot examination. Self-reported hypoglycemic episodes in the last month and hospitalization for hyperglycemia with or without DKA were also recorded.

Economic status was defined according to the Brazilian Economic Classification Criteria from the lowest to the highest estimated household income category. This classification also takes into account the educational level. For this analysis, the following classes of economic status were considered: high, middle, low and very low [22].

Patients younger than 13 years old were considered as children (toddlers, pre-scholars or scholars); patients between 13 and 18 years were deemed as adolescents [23].

The following psychosocial questions were addressed to parents on another room apart from their children/adolescents through an interview:

1. Do you think your child’s diabetes has modified the family functioning? If yes, how?

2. Did the relationship with your partner/spouse change after the diagnosis of diabetes in your child? If yes, how?

3. Does any adult (mother/father/grandparent/etc.) participate in the patient’s care? Which one?

4. Is the child with diabetes treated differently because of the disease? If yes, in which way?

5. Do you set prohibitions to your child because he/she has diabetes? If yes, in which situations?

6. Is the patient encouraged to perform diabetes management tasks?

7. Are you worried about your child’s long-term health problems?

8. Do you worry about your child having hypoglycemia?

9. Are you afraid about the development of diabetes in another child of yours?

10. Have you already checked your non-diabetic children’s blood glucose levels?

11. Do you feel overwhelmed by caring for your child’s diabetes?

12. Has your child with diabetes already slept over?

13. Do you have difficulties to set limits to your child with diabetes?

Parents’ health-related quality of life (HRQOL) indices were measured using a descriptive instrument (EQ-5D) and a visual analogue scale (EQ-VAS). EQ-5D was designed to assess five different health aspects based on the participants’ perceptions: 1) Mobility, 2) Self-care, 3) Usual activities, 4) Discomfort and 5) Anxiety/depression. EQ-VAS was designed to evaluate patients’ perceived health status using a 100-point analogue scale (0=worst perceived health; 100=best perceived health) [24,25].

In the present paper data from 1,079 parents and their children/ adolescents with T1D up to 18 years old were analyzed.

All participant institutions had the study protocol approved by local Ethics Committees. An informed consent was obtained from all participant subjects.

### Statistical analysis

The sample size of the main study was defined with respect to the number of patients with T1D living in each geographical region of Brazil, which was established using the country’s population density (38.8%, 31.7%, 23.0% and 6.6% in the southeast, north/northeast, south and mid-west regions, respectively) as described in the 2000 Brazilian Institute of Geography and Statistics’ Census (IBGE) [26], and the estimated number of patients with diabetes living in each one of these regions based on the only survey performed in Brazil in 1988 [27]. In that study, the estimated prevalence of diabetes was 7.6% in patients aged 30 to 69 years old, and the prevalence of self-reported diabetes in subjects younger than 30 years was 0.1%, the majority of which was presumed to have T1D. The compliance of each region of the country with its target enrollment was >95%.

Categorical variables were described as frequency (percentage). Numerical variables with a normal distribution were described as mean (standard deviation); those without a normal distribution were expressed by median (interquartile range). Chi-square tests (categorical variables), and t tests and ANOVA (numerical variables) were used when indicated.

Inferences were represented by hypothesis tests with a bilateral significance level of 0.05. When multiple comparisons were done, the Scheffè’s *post hoc* test was employed.

Data were stored and processed with Epiinfo 2000 and analyzed with the SPSS 11.0 statistical package.

## Results

### Demographic and socioeconomic status data of the studied parents and patients

Data were obtained from 1,079 parents (115 fathers and 964 mothers), from 1,079 T1D patients with T1D. Mothers and fathers did not differ with regard to the current age of their children (p=0.2) nor with the duration of diabetes (p=0.9).

The demographic and socioeconomic status data of the studied parents are described in Table 1. The majority (n=782; 72.5%) of families belonged to low and very low socioeconomic levels. Among the mothers, 395 (41%) were housewives and 436 (45.2%) were active workers. Considering the fathers, the majority were active workers (n=90; 78.2%). The comparison between mothers and fathers showed that mothers were younger (38.3 ± 7.5y *vs.* 41.5 ± 7.2y, respectively; p<0.001) and had attended a similar mean school-years (9.6 ± 5.9y *vs.* 10.1 ± 5.0y, respectively; p=0.3).

**Table 1:** Demographic and socioeconomic status data of the studied parents (1,079 parents)

**Variable**	
Age, years	38.6 ± 7.6
Gender, Female n (%)	964 (89.3%)
**Ethnicity, n (%)**	
Caucasian	566 (52.5%)
Non-Caucasian*	513 (47.5%)
**Economic status**	
High	72 (6.7%)
Medium	225 (20.9%)
Low	327 (30.3%)
Very low	455 (42.2%)
**Geographic region (%)**	
Southeast	346 (32.1%)
North/northeast	422 (39.1%)
South	250 (23.2%)
Mid-west	61 (5.7%)

The majority of patients (n=706; 65.4%) were seen at tertiary care level health services and (Table 2) had been followed at that center for at least one year (n=879; 81.5%). Overall, 603 patients (55.9%) were children and 476 (44.1%) were adolescents.

**Table 2:** Clinical and demographic data of the studied patients (1,079 patients)

**Variable**	
**Age, years**	11.4 ± 3.9
**Ethnicity, n (%)**	
Caucasian	566 (52.5%)
Non-Caucasian*	513 (47.5%)
**Gender, Female n (%)**	573 (53.1%)
**Age at diagnosis, years**	6.9 ± 3.9
**Age at diagnosis, years n (%)**	
0-4.9	353 (32.7%)
5-9.9	412 (38.2%)
10-14.9	282 (26.1%)
15 or more	32 (3.0%)
**HbA1c (%)**	9.2 ± 2.3%
**Duration of diabetes, years**	4.5 ± 3.5
**Levels of care, n (%)**	
Secondary	373 (34.6%)
Tertiary	706 (65.4%)
**Medical follow-up at the participating center,y**	3.6 ± 3.0
**Diabetes treatment, n (%)**	
**Insulin**	
Intermediate or long-acting	170 (15.8%)
Intermediate or long-acting plus short acting	904 (83.8%)
Insulin Pump	5 (0.4%)
Long-acting shots ≥ 3/day, n (%)	439 (40.7%)
Short-acting shots ≥ 3 /day, n (%)	591 (54.8%)
**SMBG, n (%)**	1,015 (94.1%)
**SMBG/day**	3.4 ± 1.6
**Hypoglycemia, n (%)**	610 (56.5%)
Hospitalization, n (%) **	190 (17.9%)
**Clinical visits last year**	4.3 ± 1.7

Data are presented as means, SD and n (%); SMBG; self-monitoring of blood glucose;

* African-Brazilians, Mulattos, Asians, Native Indians.

**Hospitalization by hyperglycemia with or without ketoacidosis.

### Clinical and diabetes management of the patients

The majority of the patients had been checked in 3 or more clinical visits in the prior year (n=927; 85.9%), were using combined insulin therapy (n=904; 83.8%) and used to perform 3 or more SMBG daily (n=738; 68.4%). Children had lower HbA1c levels than adolescents (8.7 ± 1.9% *vs.* 9.8 ± 2.7%, respectively; p<0.001). More children (n=150; 29.6%) than adolescents (n=78; 19.4%) achieved the goal for HbA1c (p=0<0.001). More children (n=340; 55.7%) than adolescents (n=270; 44.3%) referred hypoglycemic episodes (p=0.02) in the last month. No difference was observed in hospitalization for hyperglycemia with or without DKA between children and adolescents (Table 2).

### Impact of diabetes on family functioning and child-rearing practices from parents’ point of view

Table 3 shows parents’ answers to the main psychosocial questions. There was a frequency of missing answers that varied from 4 (0.4%) to 61 (5.7%). For this reason, data are presented in terms of both frequency and percentage.

**Table 3:** Number and percentage of parents' answers to questions focusing psychosocial aspects related to their children’s diabetes

**Parents assertions**	**n (%)**
Family functioning was modified by my child with diabetes	842 (78.5%)
After the diagnosis the relationship with my spouse changed	324 (31.6%)
There is always an adult (mother/father/grandparent/etc.) involved in diabetes care	1,041 (96.8%)
I do not treat my child with diabetes differently from my other children	781 (76.3%)
I do not set prohibitions to my child because he/she has diabetes	741 (69.1%)
The patient is encouraged to perform diabetes management tasks	978 (91.7%)
I am worried about diabetes complications	1,032 (96.4%)
I am worried about hypoglycemic episodes	1,005 (93.9%)
I’m afraid that diabetes develops in another sibling	706 (69.1%)
I have already checked my non-diabetic children’s blood glucose	654 (64.2%)
I feel overwhelmed with caring for my child’s diabetes	672 (62.8%)
My child has already slept over	440 (41.0%)
I have had difficulties in setting limits for my child with diabetes	555 (52.0%)

Family functioning was modified by the existence of a child with T1D according to 842 (78.5%) participating parents, without difference between mothers’ and fathers’ opinions (p=0.6). The main changes mentioned were related to daily routine and meals (n=388; 38.7%), followed by the occurrence of some kind of stress (n=354; 35.3%) due to diabetes management.

Considering the conjugal relationship, 324 (31.6%) parents mentioned that diabetes had had an impact on it, with little difference in the proportion of those reporting an increase in conflicts (n=119; 12.3%) and of those mentioning an improved relationship after the diagnosis of diabetes (n=128; 13.3%).

The great majority of parents (n=1,041; 96.8%) said that an adult was always involved in the care of the child with T1D, being predominantly only the mother (n=543; 50.5%). Other caregivers referred in decreasing order were both parents (n= 373; 34.7%), only the father (n=24; 2.2%) and another relative, or a non-relative person (n=135; 12.6%).

Most participating parents said that they neither treated the patient differently from their other children (n=781; 76.3%), nor set prohibitions due to diabetes (n=741; 69.1%).

The great majority (n=978; 91.7%) of the parents encouraged their children to be engaged in diabetes management tasks, particularly after reaching adolescence (n=454; 96.4% of parents of adolescents *vs.* n=524; 87.9% of parents of children aged 12 or less; p<0.001).

Irrespective of children’s age, a large proportion of parents said that they worried about diabetes complications (n=1,032; 96.4%) and about hypoglycemic episodes (n=1,005; 93.9%). The majority (n=706; 69.1%) declared to fear of another sibling developing diabetes. Almost the same proportion of mothers and fathers reported to have already checked their non-diabetic children’s blood glucose levels (n=582; 64.2% *vs.* n=72; 64.3%, respectively; p=0.9). Most parents declared to feel overwhelmed by their children’s diabetes care (n=672; 62.8%) and this feeling was significantly less frequent in parents of adolescents (n= 272; 57.7%) compared to parents of children (n=400; 66.8%) (p=0.002).

Less than half of the parents (n=440; 41%) reported that their children had already slept over. The permission to sleep over was significantly more frequent for adolescents (n=238; 50.3%) than for children (n=202; 33.7%) (p<0.001).

More than half of the parents (n=555; 52%) admitted to experiencing difficulties in setting limits for their children or adolescents with diabetes without difference between children and adolescents (p=0.6).

### Parents’ self-perceived health

On the EQ-VAS, parents’ self-perceived health status ranged from 0 to 100 with a median of 80. The 25^th^ and 75^th^ percentiles were 70 and 90, respectively. In this study, the median of 80 did not vary according to participants’ age differently from normative data - pooled from participants of 15 countries – that point a decrease in EQ VAS ratings with increasing age [25].

When analyzing the frequency of reported level 2 and level 3 problems for each of the 5 EQ-5D (Table 4) data revealed that few parents complained about health self problems, except when the items focused on discomfort (n=385; 36.0%) or anxiety/depression (n=547; 51.2%). A significantly greater proportion of mothers than fathers reported discomfort (n=356; 37.3% *vs.* n=29; 25.7%; p=0.05) and complained about anxiety/depression (n=510; 53.4% *vs.* n=37; 32.7%; p=<0.001). The complaints about anxiety/depression were not different between parents of children or adolescents (p=0.8). These complaints seem not to be influenced by socioeconomic status (p=0.2 and p=0.6 for complaints about discomfort and anxiety/depression, respectively), neither by the duration of diabetes (p=0.9 and p=0.2 for complaints about discomfort and anxiety/depression, respectively).

**Table 4:** Number and percentage of parental answers reporting problems in the five health aspects of the EQ-5D questionnaire

**I do have problems with**	**Fathers+Mothers**	**Fathers**	**Mothers**	**P-Value**
	**n (%)**	**n (%)**	**n (%)**	
Mobility	77 (7.2%)	10 (8.8%)	67 (7.0%)	0.47
Self-care	19 (1.8%)	2 (1.8%)	17 (1.8%)	0.99
Usual activities	73 (6.8%)	7 (6.2%)	66 (6.9%)	0.78
Discomfort	385 (36.0%)	29 (25.7%)	356 (37.3%)	0.05
Anxiety/depression	547 (51.2%)	37 (32.7%)	510 (53.4%)	<0.001

### Relations between psychosocial variables and patients’ glycemic control

Data showed that youth whose parents did not report difficulties in setting limits to them had significantly lower HbA1c levels (8.8 ± 2.1%) in comparison with those whose parents reported this kind of difficulty (9.7 ± 2.5%) (p< 0.001).

Although it could be detected a significant association between the statement of treating the child with diabetes differently and of having difficulties to set limits to him/her (p<0.05), 70.3% of the parents that reported to have these difficulties said they do not treat the child with diabetes in a different manner compared to his/her siblings.

The comparison of HbA1c levels of children/adolescents whose diabetes management was supervised by only their fathers (9.6 ± 2.9%), by only their mothers (9.4 ± 2.6%) or by both parents (9.1 ± 2.1%) indicates a slight tendency without a statistically significant difference for a better metabolic control when both parents were involved in the treatment (p=0.2).

Regarding parents’ well-being, we found an association between parents’ report of overwhelming due to diabetes care and anxiety/depression, as measured by the EQ-5D questionnaire (p<0.001).

Parents of patients who reported the occurrence of hypoglycemic episodes in the last month complained significantly more about anxiety/depression (n=334; 55.1%) than parents from patients who did not report it (n=189; 45.7%), (p=0.02).

Also a significantly greater proportion of parents of youth who had been hospitalized due to hyperglycemia reported anxiety/depression than those whose children had not been hospitalized (98 out of 167; 58.7% *vs.* 439 out of 881; 49.8%, respectively; p=0.04)

## Discussion

This study showed that parents of youths with T1D consider that diabetes introduces several modifications in the family lifestyle. Other studies about psychological experience of parents of children with T1D revealed that family life revolves around diabetes, with a constant focus on meals, blood glucose levels monitoring, and insulin administration requiring parents to devote considerable time and effort to develop a new routine [28]. Parents frequently express the need for constant vigilance to determine the meaning of their child behaviors that could be indicative of hypoglycemia or hyperglycemia [29]. In this study we verified that parents frequently worried about their child’s long-term health problems and hypoglycemic episodes in consonance with findings of other studies [18,30]. Our data also showed an association between the occurrence of hypoglycemic episodes in the last month reported by patients and parents’ complaints about anxiety/depression.

It is well established that the need for tight blood glucose control presents additional stress to young people and their families [31], especially if they are unable to reach and maintain a good glycemic control [32]. In the present study, parents - especially mothers - reported discomfort and anxiety/depression, mainly if their children/adolescents had experienced hypoglycemic episodes in the last month or if they had been hospitalized due to hyperglycemia or diabetic ketoacidosis, independently of socioeconomic status. The reason for the observed difference between mothers and fathers is probably the fact that more mothers than fathers take care of their children/adolescents with T1D.

Although the general scores of quality of life self-assessed by participating parents through EQ VAS tended to be similar to normative data [25], parents complained about feeling overwhelmed by caring for their children’s diabetes similarly to those described in other studies [18]. Moreover, in the present study parents also reported a decrease in distress related to diabetes care over time [33,34].

Our data showed a worsening of metabolic control during adolescence in consonance to data found in the literature [35-37]. In this study, parents encouraged their children to be engaged in diabetes management tasks, particularly after reaching adolescence. Although continued involvement of parents in treatment management is associated with better health and psychosocial outcomes in youth with T1D [38-40], the level and type of parental involvement change with the child’s developmental stage. As children reach adolescence, the parental involvement in the treatment may conflict with the developmental task of increasing autonomy that adolescents have. Several studies have found that when parents give up responsibility for treatment management too early, adolescents have poorer adherence and deteriorate their glycemic control [10,40]. This seems to occur when patients take on responsibility for their diabetes management when they still do not have the maturity to handle it [40]. Therefore, parents should be encouraged to maintain continued involvement in treatment management throughout adolescence, and to transfer responsibility to their children only after they have demonstrated to be able to assume and perform adequately diabetes management tasks. It is also important to consider that not all involvement is beneficial; over-involvement or intrusive parenting may have a negative impact on adolescents’ adaptation [8,39,41-45]. The great challenge is to reach the equilibrium between parents’ involvement and patients’ independence.

Moreover, in the present study many parents admitted experiencing difficulties in setting limits for their children and this parenting style was associated with poorer metabolic control. Child development experts have highlighted the importance of three factors in parenting children and adolescents: control, involvement, and affection [46]. Clearly, children and adolescents need all three factors throughout their youth.

Although all children need high levels of affection and involvement, these should be delivered differently to successive age ranges. In the case of diabetes, younger children may need a great deal of parental involvement in the physical aspects of caring for their diabetes (e.g., giving shots, drawing up insulin), whereas older children may need verbal prompts and cues to facilitate self-management behaviors.

Parents need to set limits and have clear expectations for the child with diabetes as they would do with a child without diabetes. Unfortunately, feelings of guilt or pity about the child’s disease may interfere with such limit-setting [47,48] with prejudicial consequences to the metabolic control of a child with T1D [49,50].

Glycemic control (as assessed by HbA1c) of our patients cared by both parents tended to be better than that of those cared by only one of them. Mothers are still the primary caregivers for children with diabetes [51]. However, fathers play a crucial role in general adolescence [52] and their support [53] and monitoring [54] may be associated with better diabetes management, mainly if the young person and his/her family work together as a team [55]. Other studies suggest that diabetic youths who live with single parents are in poorer metabolic control than those raised in two-parent families [56,57].

Many participating parents declared to have fear of another sibling developing diabetes and reported to have already checked their non-diabetic children’s blood glucose levels. A number of studies have examined the T1D risk perceptions in populations with and without a first degree relative with T1D. Family members of patients with T1D seem to be aware that they are at increased risk for the disease and this can be a source of stress [58,59]. At least two other studies verified that most parents of at-risk children acknowledge some worry or concern when they learn a beloved one is at increased risk for developing T1D [60,61].

The principal strength of our data is the population-based ascertainment of diabetes cases in a widely diverse young Brazilian population and the resulting large sample of patients with T1D. The present study included patients and parents belonging to a wide range of ethnic groups from all geographic regions of the country, and the data collection process followed a uniform and standardized protocol in all participating centers. To our knowledge this is the first study conducted in all geographic regions of Brazil addressing the impact of T1D on family functioning and child-rearing practices according to parents’ perception, evaluating parents’ health-related quality of life and investigating the relations between psychosocial variables and the glycemic control of young patients with T1D.

Finally, some limitations of the main study must be addressed especially those related to the sample's characteristics. All patients received health care in public care clinics and lived in large Brazilian cities. Patients receiving care in private clinics and in primary care level could have been missed, but the former group probably represents the minority of patients with T1D receiving treatment in Brazil.

Regarding the evaluation of parents’ health-related quality of life, the lack of a control group of parents of non-diabetic children/adolescents represents another limitation of this study. The use of non-standardized psychosocial questions addressed to parents constituted another limitation, although the majority of these questions were tested by the first author on a previous study that assessed the effect of a psycho-educational program on T1D patients and their parents [62].

Moreover, all data concerning psychosocial variables and the occurrence of hypoglycemic episodes/hospitalization were obtained through parents’ and patients’ self-report, respectively.

## Conclusions

After the diagnosis of T1D, the lifestyle of all family members changes and this interferes in their quality of life.

Mothers are still the primary caregivers for children/adolescents with T1D although patients cared by both parents tended to have better metabolic control.

Parents need to set limits for the child/adolescent with diabetes as they would for a child/adolescent without diabetes. Difficulty to set limits for children/adolescents may be a risk for poor diabetes control. Therefore parent education should be recommended.

This study demonstrates the importance to consider young patients’ adjustment to diabetes in the context of their family environment. The specific needs of T1D patients and their impact on family life must be considered for future improvement on therapy elements and strategies.

## Endnote

^a^The public secondary care level centers are those that follow ambulatory outpatients and the public tertiary care level clinics follow ambulatory outpatient clinics in university hospitals.

## Abbreviations

BrazDiab1SG: Brazilian Type 1 Diabetes Study Group; BDS: Brazilian Diabetes Society; CNPq: Brazilian National Counsel of Technological and Scientific Development; DCCT: Diabetes Control and Complications Trial; DKA: Diabetic ketoacidosis; EDIC: Epidemiology of Diabetes Interventions and Complications Trial; HbA_1_c: Glycated hemoglobin; HRQOL: Health-related quality of life; IBGE: Brazilian Institute of Geography and Statistics; NBHCS: National Brazilian Health Care System; QoL: Quality of Life; SMBG: Self-monitoring of blood glucose; T1D: Type 1 diabetes.

## Competing interest

The authors of this manuscript do not have any conflict of interest.

## Authors' contributions

All authors participated in the development of this manuscript. All authors also read and approved the final manuscript.

## Authors' information

Brazilian Type 1 Diabetes Study Group (BrazDiab1SG)

Executive steering committee: Marilia Brito Gomes (chair), Roberta Cobas, Sergio Atala Dib, Carlos Negrato.

Principal investigators are indicated by an asterisk. Program coordinators are underlined.

**Department of Internal Medicine, Diabetes Unit, State University of Rio de Janeiro, Brazil Universidade Estado Rio de Janeiro:** Marilia de Brito Gomes*, M.D. (mariliabgomes@gmail.com), Roberta Arnoldi Cobas, M.D. (robertacobas@gmail.com), Alessandra Saldanha de Mattos Matheus, M.D (alessandramatheus79@yahoo.com), Lucianne Righeti Monteiro Tannus, M.D. (luciannetannus@ig.com.br)

**Federal University Hospital of Rio de Janeiro:** Lenita Zajdenverg*, M.D. (lenitazaj@gmail.com), Melanie Rodacki M.D. (mrodacki2001@yahoo.com.br)

General Hospital of Bonsucesso: Neuza Braga Campos de Araújo* M.D. (russarj@terra.com.br), Marilena de Menezes Cordeiro M.D. (marmecor@gmail.com)

**University Hospital Clementino Fraga Filho – Children Institute Martagão Teixeira:** Jorge Luiz Luescher* M.D.(luescher_@hotmail.com), Renata Szundy Berardo M.D. (rszundy@br.inter.net)

**Diabetes Unit, University Hospital of São Paulo, São Paulo:** Marcia Nery* , M.D. (marcianery@hcnet.usp.br), Catarina Cani, M.D. (catarinagcani@gmail.com), Maria do Carmo Arruda Marques, M.D. (mcarruda@hcnet.usp.br)

**Pediatric Unit of Endocrinology - Santa Casa Hospital, São Paulo:** Luiz Eduardo Calliari* , M.D. (calliari.cidep@uol.com.br), Renata Maria de Noronha, M.D. (renata_noronha@uol.com.br)

**Children Institute of Endocrinology, University Hospital of São Paulo, São Paulo:** Thais Della Manna* , M.D. (thais.manna@icr.usp.br), Roberta Salvodelli (robysds@hotmail.com), M.D., Fernanda Garcia Penha, M.D. (fepenha@ajato.com.br)

**Ribeirão Preto Medical School of São Paulo University, Sao Paulo:** Milton Cesar Foss*, M.D. (mcfoss@fmrp.usp.br), Maria Cristina Foss-Freitas, M.D. (mcfoss@fmrp.usp.br)

**Department of Internal Medicine, Medical School, State University of São José do Rio Preto:** Antonio Carlos Pires*, M.D.(fpires@terra.com.br), Fernando Cesar Robles, M.D.(roblesmed@ig.com.br)

**Bauru’s Diabetics Association, Bauru, São Paulo:** Carlos Antonio Negrato* , M.D. (carlosnegrato@uol.com.br), Maria de Fatima Guedes, M.D. (tatiguedeses@hotmail.com)

**Diabetes Unit, Federal University of São Paulo State, São Paulo:** Sergio Atala Dib*, M.D. (sergio.dib@unifesp.br), Patricia Dualib, M.D. (patricia.dualib@uol.com.br)

**Endocrinology Unit, Hospital of Santa Casa of Belo Horizonte, Minas Gerais:** Saulo Cavalcanti da Silva*, M.D. (scsendocrino@yahoo.com.br), Janice Sepúlveda, M.D. (janicesepulveda@terra.com.br)

**Diabetes Unit, State University Hospital of Londrina, Paraná:** Henriqueta Guidio de Almeida*, M.D. (henriqueta@uel.br), Emerson Sampaio, M.D. (emersamp@hotmail.com)

**Hospital de Clínicas da Universidade Federal do Paraná:** Rosangela Roginski Rea*, M.D. (rosangelarea@uol.com.br), Ana Cristina Ravazzani de Almeida Faria, M.D. (aravazzani@uol.com.br)

**Institute of Diabetic Children, Rio Grande Sul:** Balduino Tschiedel*, M.D. (badutsch@gmail.com) , Suzana Lavigne, M.D. (suzanalavigne@yahoo.com.br), Gustavo Adolfo Cardozo, M.D. (byguga@hotmail.com);

**Clinical Hospital of Porto Alegre, Rio Grande do Sul:** Mirela Azevedo*, M.D. (mirelajobimazevedo@gmail.com), Luis Henrique Canani, M.D. (luishenriquecanani@gmail.com), Alessandra Teixeira Zucatti, M.D. (alezucatti@hotmail.com);

**University Hospital of Santa Catarina:** Marisa Helena Cesar Coral*, M.D.

(marisahcc@uol.com.br), Daniela Aline Pereira, M.D. (danialine@yahoo.com)

**Endocrinology and Diabetes Institute of Joinville, Santa Catarina:** Luiz Antonio de Araujo*, M.D. (luiz@endoville.com.br)

**General Hospital of Goiânia:** Nelson Rassi*, M.D. (nrassi@brturbo.com.br), Leticia Bretones de Araujo, M.D. (leticiabretones@yahoo.com.br)

**Diabetes and Endocrinology Center of Bahia:** Reine Marie Chaves Fonseca*, M.D. (reinemar@terra.com.br); Alexis Dourado Guedes, M.D. (dr.alexis@uol.com.br), Odelisa Silva de Mattos, M.D. (odelisam@yahoo.com.br)

**Federal University of Maranhão:** Manuel Faria*, M.D. (mfaria@inlab.com.br), Rossana Azulay, M.D. (rossanaendocrino@gmail.com)

**Diabetes and Hypertension Center of Ceará:** Adriana Costa e Forti*, M.D. (adrianaforti@uol.com.br), Cristina Façanha, M.D. (crisffacanha@hotmail.com)

**Federal University of Ceará:** Renan Montenegro Junior*, M.D. (renanjr@ufc.br), Ana Paula Montenegro, M.D. (clinicarenanmontenegro@hotmail.com)

**Federal University of Sergipe:** Naira Horta Melo*, M.D. (nhmelo@gmail.com), Karla Freire Rezende, M.D. (kfr@infonet.com.br)

**Federal University Hospital of Campina Grande, Paraíba:** Alberto Ramos*, M.D. (ajsr@uol.com.br)

**Federal University Hospital of Pará:** João Soares Felício*, M.D. (felicio.bel@terra.com.br), Flavia Marques Santos, M.D. (drafms@bol.com.br)

**Getúlio Vargas University Hospital of Amazonas, Adriano Jorge Hospital:** Deborah Laredo Jezini*, M.D. (dljezini@hotmail.com)

**Regional Hospital of Taguatinga, Brasília:** Hermelinda Cordeiro Pedrosa*, M.D (pedrosa.hc@globo.com), Monica Tolentino, M.D. (monicatolentino@uol.com.br); Flaviene Alves Prado, M.D. (secretariadraflaviene@gmail.com)

## Funding

The main research received support from CNPq (Brazilian National Counsel of Technological and Scientific Development).
